# Genomic landscape and fine-scale population structure of *Helicobacter pylori* across China

**DOI:** 10.1186/s40364-026-00932-0

**Published:** 2026-05-16

**Authors:** Yi Dou, Pengfei Kong, Mingzhu Huang, Yonghu Xu, Jingyu Guo, Yong Xie, Xiaohua Jiang, Yantao Duan, Gonghong Wei, Dazhi Xu

**Affiliations:** 1 Department of Gastric Surgery, Fudan University Shanghai Cancer Center, Shanghai, China; 2 Department of Oncology, Shanghai Medical College, Fudan University, Shanghai, China; 3 Department of Oncology, Fudan University Shanghai Cancer Center, Shanghai, China; 4 Department of Gastroenterology, Digestive Disease Hospital, The First Affiliated Hospital of Nanchang University, Jiangxi Medical College, Nanchang University, Nanchang, Jiangxi Province China; 5 Department of Gastric Surgery, Shanghai Dongfang Hospital, Shanghai, China; 6 Department of Biochemistry and Molecular Biology, School of Basic Medical Sciences, Fudan University Shanghai Cancer Center, Shanghai Medical College of Fudan University, Shanghai, China

**Keywords:** *Helicobacter pylori*, Gastric cancer, Population structure, Antibiotic resistance

## Abstract

**Background:**

East Asian *Helicobacter pylori* (*H. pylori*) strains are commonly classified as a single hspEAsia lineage characterized by elevated virulence. However, gastric cancer incidence varies markedly across China, suggesting that clinically relevant bacterial heterogeneity may exist within this framework. A systematic assessment of fine-scale population structure and its functional correlates in Chinese *H. pylori* remained limited.

**Methods:**

We analyzed whole-genome sequencing data from 1,243 *H. pylori* isolates collected from 20 provinces and regions across China, including 50 newly sequenced clinical strains from Shanghai. Fine-scale population structure was resolved using coancestry–based clustering and chromosome painting. Subpopulations were further characterized by pangenome composition, virulence factor repertoires, genome-wide fixation index (Fst), and predicted antibiotic resistance–associated mutations. E-test minimum inhibitory concentration (MIC) assays were performed to compare phenotypic susceptibility with mutation-based resistance prediction.

**Results:**

Six geographically structured subpopulations were identified within Chinese hspEAsia. SubtypeCentral represented a widely distributed mainland lineage, whereas subpopulations from Inner Mongolia and Taiwan showed the greatest genetic divergence. Chromosome painting revealed strong within-lineage ancestry cohesion in SubtypeTaiwan, contrasted by extensive admixture in Inner Mongolia and Yunnan. Recurrent high-Fst loci across subpopulations, including *glnA*, *frpB4*, and *HP1501*, highlighted genomic regions contributing disproportionately to population differentiation. Marked heterogeneity in virulence profiles was observed. SubtypeInnerMongolia showed a higher prevalence of *cagA*-negative or Western-type *cagA* variants and a reduced overall repertoire of virulence genes. Predicted antibiotic resistance patterns were also strongly subtype dependent. Notably, SubtypeTaiwan exhibited an exceptionally high rifampicin resistance rate driven almost exclusively by a single *rpoB* A2414V mutation. E-test validation in the newly collected isolates provided supportive phenotypic evidence for the mutation-based resistance strategy.

**Conclusions:**

Chinese *H. pylori* hspEAsia strains comprise multiple regionally structured subpopulations with distinct evolutionary histories, gene content, virulence profiles, and predicted resistance determinants. This fine-scale genomic classification provides a biological basis for understanding regional disparities in gastric cancer risk and genotypic resistance, and supports the need for subtype-aware surveillance and region-specific clinical management strategies in China.

**Supplementary Information:**

The online version contains supplementary material available at 10.1186/s40364-026-00932-0.

## Background

*Helicobacter pylori (H. pylori)* has accompanied human populations for thousands of years, and its evolutionary history closely parallels human migration [1]. On a global scale, population genetic frameworks based on multi-locus sequence typing and whole-genome sequencing have delineated several major *H. pylori* lineages, including hpAfrica1, hpAfrica2, hpNEAfrica, hpEurope, hpAsia2, hpSahul and hpEastAsia [2]. In East Asia, the hspEAsia lineage dominates and is notorious for its high virulence, linked to the heavy burden of gastric cancer [3]. This issue is particularly relevant in China, where *H. pylori* prevalence has declined over time but is still high, at approximately 37%–42.8% in 2014–2023, based on recent national meta-analyses [4, 5]. This association is commonly attributed to the high prevalence of virulence-associated genotypes, particularly East Asian–type cagA carrying the EPIYA-ABD motif and the vacA s1m1 genotype, which are more frequent than in Western populations [6, 7].

However, although the hspEAsia subgroup is considered relatively homogeneous, there are significant regional variations in gastric cancer incidence within East Asia. Recent genome-wide analyses have begun to challenge this assumption. HspEAsia can be subdivided into geographically associated subgroups, which differ in ancestry composition and exhibit genetic divergence related to host interaction [8]. These findings suggest that substantial within-lineage diversity exists within what was previously considered a single lineage, warranting further evaluation of its possible clinical relevance. China provides a particularly informative setting for studying *H. pylori* population structure because of its large geographic span, heterogeneous host backgrounds, and marked variation in environment and diet. However, detailed characterization of *H. pylori* population structure within China remains incomplete. Previous studies focused on pan–East Asian datasets or on limited regions within China [9]. Some studies focus solely on specific high-risk regions or particular ethnic groups. For instance, strains from the gastric cancer hotspot in Shandong were found to form a relatively independent genetic cluster [10], while strains from Tibet exhibited unexpected associations with the hpEurope lineage [11]. These observations imply the presence of underrecognized subtypes shaped by regional history and local adaptation.

Beyond geographical isolation, selective pressure from antibiotics has also been a significant driving force in the contemporary evolution of *H. pylori*. The widespread use of eradication therapy in China has been accompanied by rising resistance rates to commonly used antibiotics, including clarithromycin, metronidazole, and levofloxacin [12]. Whole-genome sequencing allows precise detection of classical resistance-associated mutations, such as A2143G in 23S rRNA and substitutions at positions N87 or D91 in gyrA [13, 14]. At the same time, genome-wide data revealed additional resistance-related changes, including variation in efflux pump genes and other loci that show regional differentiation [10, 15]. These resistance traits may not evolve independently of population structure [16] but instead interact with lineage history and local selective pressures.

In summary, although hspEAsia is the predominant strain circulating in China, significant variations in infection rates and gastric cancer incidence across different regions necessitate systematic analysis based on large-scale data. To address this gap, this study systematically analyzed whole-genome sequencing data from 1,243 clinical *H. pylori* isolates sourced from 20 provinces and regions across China, including 50 new strains clinically isolated from Shanghai. By reconstructing population structure at fine scale, we identify geographically structured *H. pylori* subpopulations within China and characterize subtype-associated differences in ancestry, pangenome composition, virulence-associated genes, and predicted resistance determinants. This work provides a genomic framework for future studies to assess whether region-associated population structure can inform surveillance, targeted molecular assays, and risk stratification.

## Methods

### Bacterial culture and genome sequencing

Twenty-four strains were derived from patients who underwent surgery at Fudan University Shanghai Cancer Center (FUSCC) between January 2023 and January 2025, and 26 strains were isolated from endoscopic mucosal samples from non-gastric cancer patients at FUSCC and Dongfang Hospital of Tongji University. *H. pylori* isolates were obtained through standard culturing procedures. The strains were cultured on Brain Heart Infusion agar (Oxoid, CM1136) supplemented with 5% sterile defibrinated horse blood (Baibo, China) and 1% *H. pylori*-selective supplement (Oxoid, SR0147E), incubated at 37 °C under microaerophilic conditions for 3–5 days. Genomic DNA was isolated from individual colony isolates using the QIAmp DNA Mini Kit (QIAGEN, #51804). DNA quality was evaluated using a NanoDrop2000 spectrophotometer. Whole genome sequencing services for *H. pylori* were provided by Majorbio. The standard Illumina TruSeq protocol was employed for library preparation, as per the Illumina TruSeq DNA Sample Preparation Guide. The samples underwent quality control checks, followed by library construction and sequencing as paired-end, 2 × 150 bp, resulting in high-quality genomic data stored in paired-end FASTQ format. Quality assessment was performed using FastQC v0.11.7, while AdapterRemoval v2.2.2 [17] and SOAPec v2.03 [18] were employed for quality correction. The genomic data were further assembled using SPAdes v3.12.0 [19] pipelines.

### Phenotypic antimicrobial susceptibility testing

Antimicrobial susceptibility testing of newly collected clinical isolates was performed using the E-test method (BIO-KONT, Wenzhou, China). MICs were determined for amoxicillin (AML), clarithromycin (CLR), levofloxacin (LEV), metronidazole (MTZ), rifampicin (RFP), and tetracycline (TET). Bacterial suspensions were spread onto blood agar plates, followed by application of E-test strips after drying. Plates were incubated at 37 °C under microaerophilic conditions for 72 h, and MICs were subsequently recorded. Resistance was interpreted according to the latest EUCAST guidelines (www.eucast.org).

### Dataset compilation and quality control

*H. pylori* genomes from various provinces in China, along with reference genome sequences from around the world, were obtained from NCBI and EnteroBase. All genomic assemblies underwent a rigorous quality control workflow using QUAST (v5.3.0) [20]. Low-quality genomes were excluded based on the following criteria: (i) fragmentation index > 500 contigs, or (ii) abnormal genome size deviating significantly from the species average (1.6Mbp). To mitigate sampling bias arising from epidemiologically linked outbreaks or duplicate submissions, pairwise Average Nucleotide Identity (ANI) was calculated using FastANI [21]. Strains sharing > 99.99% ANI were identified as clonal; in such cases, only one representative genome was retained for downstream population genetics analysis to minimize residual clonality. All curated genomes were uniformly re-annotated using Prokka (v1.14.6) [22] to ensure consistency in gene prediction. Gastric cancer incidence data for each province/region were obtained from the latest official reports released by regional cancer centers or disease control centers. *H. pylori* infection rates for each province were obtained from published literature.

### Fine-scale population structure and phylogenetic analysis

Single nucleotide polymorphisms (SNPs) were identified using the Snippy (https://github.com/tseemann/snippy, v4.6.0) pipeline with assembly-based SNP calling. The complete genome of *H. pylori* strain 26,695 (NC_000915.1) served as the reference for sequence alignment. The population structure was resolved using the haplotype-based fineSTRUCTURE (v4.1.0) pipeline [23]. The core VCF was filtered to retain only biallelic SNPs, and sites with any missing genotype calls were excluded. ChromoPainter (v2) was employed to infer the “chunk length” of DNA donated from one strain to another, generating a co-ancestry matrix that quantifies the shared genetic history between all strain pairs. For ancestry painting, all strains were treated as recipients, whereas donor strains were randomly selected from each subgroup (*n* = 15 per subgroup). The Markov Chain Monte Carlo (MCMC) simulation consisted of 100,000 burn-in iterations followed by 100,000 sampling iterations. Population structure was inferred using ADMIXTURE 1.3.0 [24] with a haploid model. ADMIXTURE was run for K = 1–8 with 10-fold cross-validation, and the optimal K was selected based on the lowest cross-validation error. To evaluate the impact of uneven regional sampling, we performed a balanced down-sampling sensitivity analysis by capping isolates per province at 30 and repeating fineSTRUCTURE and PCA across 10 random subsets. The final subdivision number was defined by concordance across the coancestry matrix, fineSTRUCTURE tree topology and complementary analyses (ADMIXTURE and down-sampling). Based on these criteria, K = 6 was selected for downstream analyses.

To place Chinese isolates within a global context, we further analyzed the dataset using GrafGen v1.6.0 [25], which assigns *H. pylori* genomes based on genetic distance to established reference populations, enabling comparison with previously defined groups such as hpgpAsia.

### Pangenome analysis

*H. pylori* Pangenome Analysis was performed using Panaroo (v1.3.4) [26]. The “strict” mode was employed to effectively filter out spurious gene calls and reduce graph complexity caused by fragmentation. A pan-genome–based phylogenetic tree was constructed using IQ-TREE (v2.2.2) [27]. Phylogenetic trees were visualized and annotated using the iTOL (Interactive Tree of Life) platform [28]. Genes were classified based on their frequency within the population: “core genes” were defined as those present in ≥ 99% of isolates. To investigate functional divergence between identified subpopulations, representative protein sequences were annotated using EggNOG-mapper v2 [29] against the EggNOG v5.0 database using DIAMOND algorithm v2.0.11 [30]. Functional enrichment analysis was performed based on Clusters of Orthologous Groups (COG) categories to identify adaptive traits specific to different lineages.

### Profiling of virulence factors

Putative virulence factors were screened by aligning genomic assemblies against the Virulence Factor Database (VFDB) using ABRicate (v1.0.1, https://github.com/tseemann/abricate). Genes were considered present if they met the threshold of ≥ 80% nucleotide identity and ≥ 70% sequence coverage, which are commonly used thresholds in bacterial virulence screening. Two key pathogenicity islands were subjected to detailed subtyping. The full-length *cagA* gene sequences were extracted and translated. The C-terminal variable region was scanned to identify EPIYA motifs. Strains were classified into East Asian-type (EPIYA-ABD) or Western-type (EPIYA-ABC) based on the specific amino acid sequences. The vacA signal (s), intermediate (i), and middle (m) genotypes were determined by comparing translated protein sequences against a curated library of subtype-specific consensus motifs using a reference-based similarity alignment strategy.

### Genotypic antibiotic resistance profiling

A comprehensive database of predicted resistance-conferring mutations was curated based on literature and the CARD database, targeting 23S rRNA (clarithromycin), *gyrA/gyrB* (levofloxacin), *pbp1A*,* pbp2*,* hefC and hofH* (amoxicillin), *rdxA/frxA* (metronidazole), *16S rRNA* (tetracycline), and *rpoB* (rifampicin). The genomic variants of each strain were compared against curated hotspot mutations within the GenBank reference. Resistance was predicted based on the presence of: (i) validated non-synonymous point mutations (e.g., A2143G in 23S rRNA, N87K in *gyrA*); and (ii) loss-of-function mutations (nonsense mutations or frameshifts leading to premature truncation) specifically for the nitro reductase genes *rdxA* and *frxA*. When a strain exhibits predicted resistance mutations to three or more different antibiotics, it is classified operationally as a multidrug-resistant (MDR) strain.

### Fixation index (Fst) analysis

We calculated the Fst value for each SNP by subtypes using the *site.FST* function from the PopGenome v2.7.5 package [31]. Each subtype was compared against the remaining Chinese populations. Functional annotation of significant SNPs was performed using SnpEff v 5.2 [32]. A Manhattan plot based on Fst values was generated using the ggplot2.

### Statistical analysis

For comparisons of categorical variables, including the prevalence of virulence-associated genes, predicted antibiotic resistance, and resistance-associated mutations across subpopulations, Pearson’s Chi-square test was used when expected cell counts were adequate; otherwise, Fisher’s exact test was applied. For large-scale subtype-comparison analyses of virulence-associated genes and predicted resistance-related features, P values were further adjusted for multiple testing using the Benjamini–Hochberg false discovery rate (FDR) method, and FDR-adjusted P values < 0.05 were considered statistically significant. For focused pairwise comparisons, effect sizes were reported as odds ratios (ORs) where appropriate. Continuous variables that were not normally distributed were compared using the Mann–Whitney U test.

## Results

### Genomic characteristics and virulence profiles of newly sequenced isolates

A total of 50 *H. pylori* clinical strains were successfully isolated and sequenced from patients in Shanghai, comprising 24 cases of gastric cancer (GC) and 26 cases of gastritis (Table S1). All patients were of Han ethnicity. The de novo assembly yielded high-quality draft genomes with an average size of 1.59 Mb and a mean GC content of 38.77%. The average number of contigs per assembly was 77.6, indicating high genomic continuity suitable for downstream comparative analysis. Regarding the primary virulence determinants, the cagA gene was identified in all 50 isolates (100%). Further motif analysis confirmed that all cagA-positive strains belonged to the East Asian type (EPIYA-ABD), consistent with the dominant lineage in Mainland China. The vacA allelic diversity was also characterized; excluding 6 isolates that were non-typeable due to sequence fragmentation, the remaining strains were classified into two major genotypes: s1/i1/m1 (*n* = 13; 7 from GC and 6 from gastritis) and s1/i1/m2 (*n* = 31; 13 from GC and 18 from gastritis).

### Genomic landscape of *H. pylori* in China

**Fig. 1:**
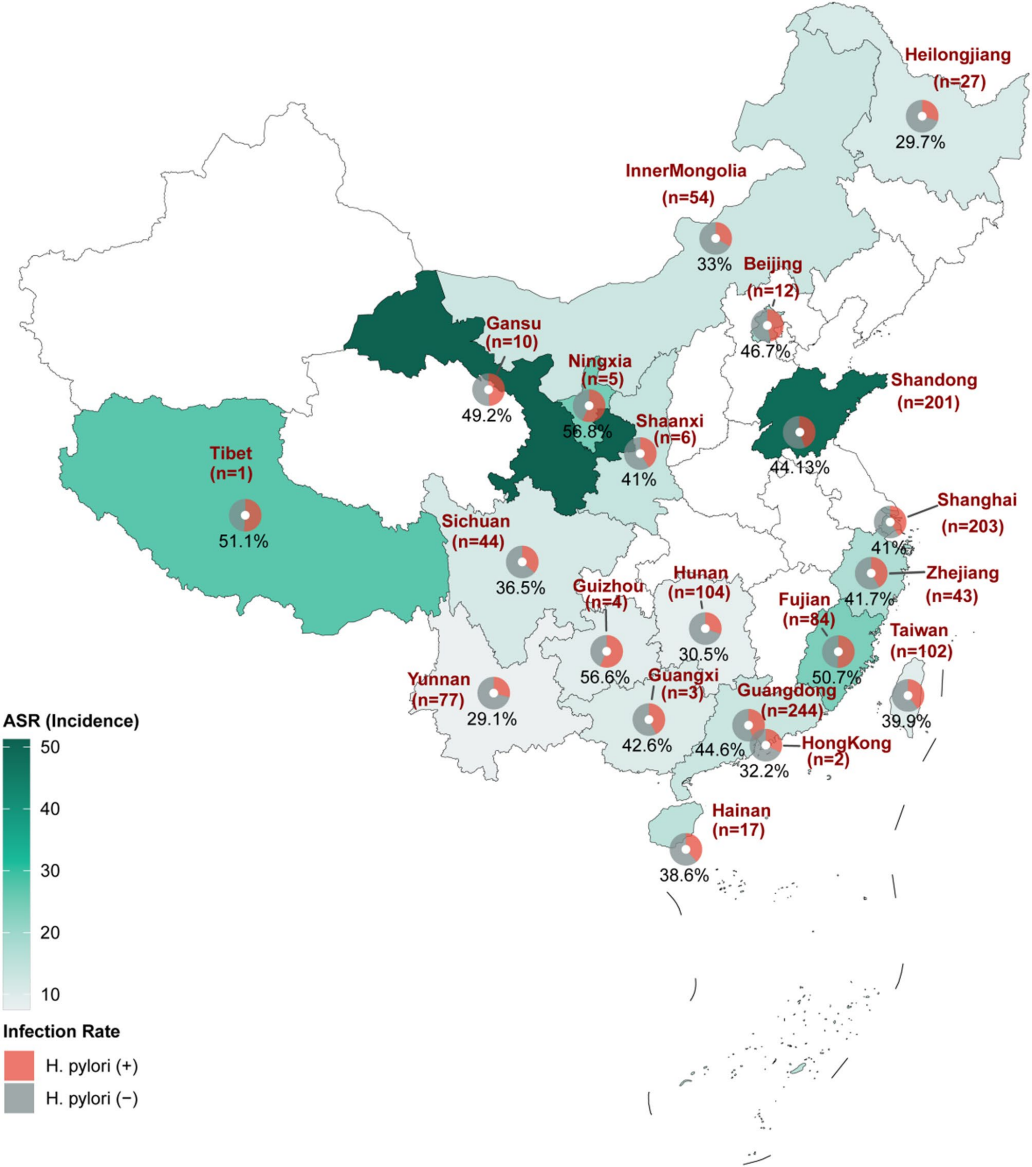
Geographic distribution of *H. pylori* strains included in this study (*n* = 1,243). A total of 1,243 Chinese clinical isolates included in the final analysis are shown according to their province or region of origin. Background color intensity reflects the age-standardized gastric cancer incidence rate (ASR) for each region, with darker colors indicating higher incidence. Pie charts summarize the reported regional *H. pylori* infection rates derived from published literature and public health reports

After quality control, a dataset comprising 1,369 *H. pylori* genomes was retained for downstream analyses (Table S2). These genomes included 126 well-characterized reference strains reported previously and 1,243 isolates collected from nearly all major geographic regions of China, providing broad national coverage (Fig. 1). East China contributed the largest proportion of isolates, with samples from Shanghai (*n* = 203), Shandong (*n* = 201), Fujian (*n* = 84), Zhejiang (*n* = 43). Southeastern coast and islands were also well represented, including Guangdong (*n* = 244), Hainan (*n* = 17), Guangxi (*n* = 3), Hong Kong (*n* = 2) and Taiwan region (*n* = 102). Additional isolates were obtained from Central China (Hunan, *n* = 104), North China (Inner Mongolia, *n* = 54; Beijing, *n* = 12), and Southwest China (Yunnan, *n* = 77; Sichuan, *n* = 44; Guizhou, *n* = 4; Tibet, *n* = 1). The inclusion of isolates from Northeast China (Heilongjiang, *n* = 27) and Northwest China (Gansu, *n* = 10; Shaanxi, *n* = 6; Ningxia, *n* = 5) ensured coverage of high-latitude and inland regions that are often underrepresented in large-scale genomic surveys.

Regional *H. pylori* infection rates were examined alongside provincial gastric cancer incidence data (Table S3). The overall infection rate across China was estimated at 41.78%. Higher infection rates were observed in eastern and southeastern coastal provinces, including Shandong (44.13%), Fujian (50.7%), and Guangdong (44.6%), as well as in parts of the northwest, such as Gansu (49.2%) and Tibet (51.1%). In contrast, lower infection rates were reported in northern regions, including Heilongjiang (29.7%) and Inner Mongolia (33.0%), and in the southwest, such as Sichuan (36.5%) and Yunnan (29.1%). Gastric cancer incidence showed marked regional variation, with the highest rates observed in Shandong and Gansu. Provinces along the southeastern coast also exhibited elevated incidence. In general, regions with higher infection rates tended to show higher gastric cancer incidence, although this relationship was not uniform across all areas.

The dataset also captures substantial ethnic diversity within China. Isolates from central and coastal regions were predominantly derived from individuals of Han ethnicity. In contrast, samples from border and high-latitude regions included a higher proportion of ethnic minorities. Among isolates from Inner Mongolia, 20 of 54 were obtained from individuals of Mongol ethnicity. The Yunnan cohort (*n* = 77) showed particularly high diversity, including isolates from Mosuo (*n* = 27), Pumi (*n* = 17), Naxi (*n* = 7), and Bai (*n* = 3) populations. Additional minority-associated isolates included Yao individuals from Guangxi (*n* = 3), Li individuals from Hunan (*n* = 2), and one Tibetan individual from Tibet. This combination of broad geographic coverage and diverse host backgrounds provides a solid basis for examining fine-scale population structure and for exploring how host population history may have shaped *H. pylori* diversity within China.

### Fine-scale population stratification

**Fig. 2:**
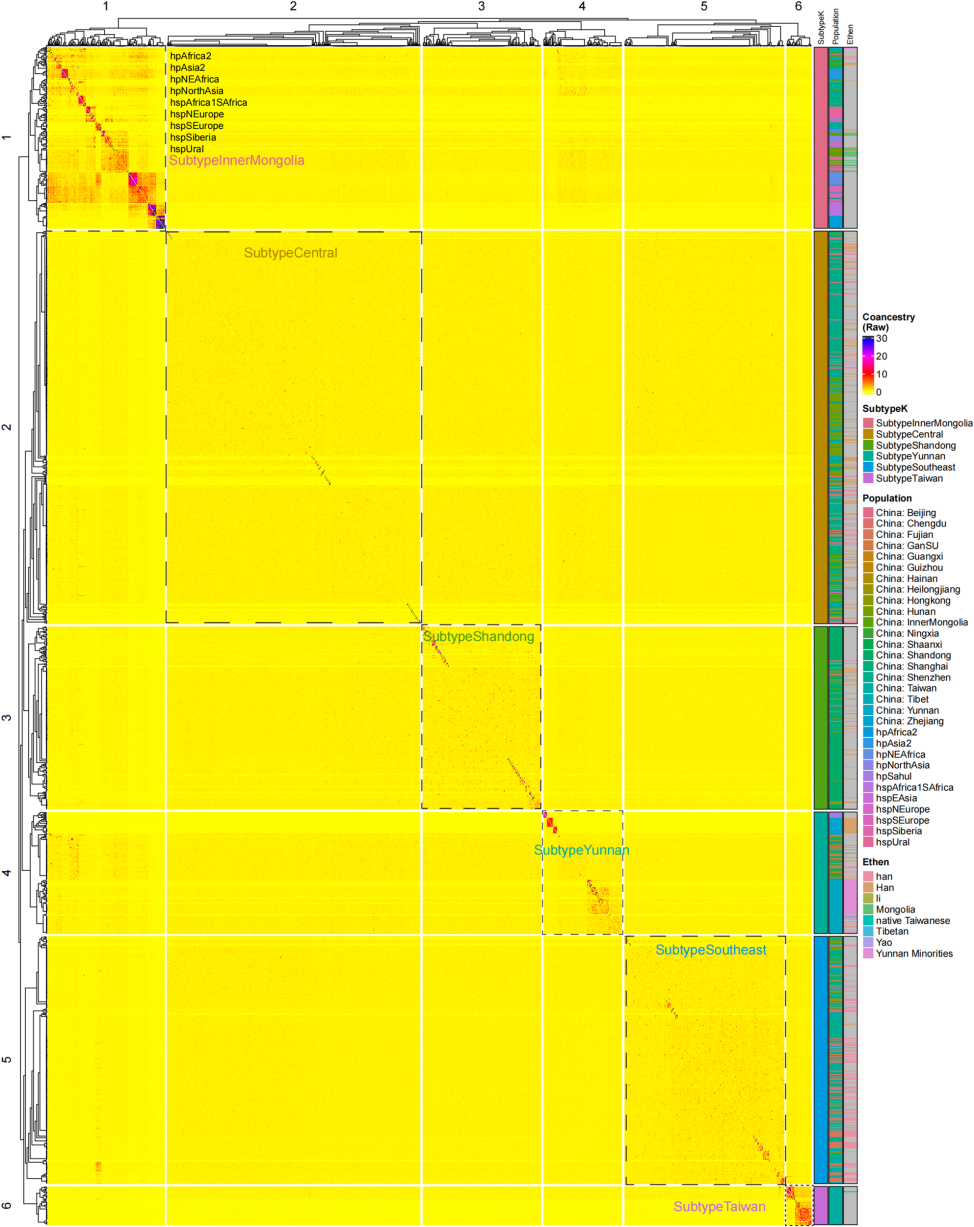
Co-ancestry matrix and phylogenetic relationships of *H. pylori* strains included in this study. The heatmap shows the pairwise co-ancestry matrix inferred by ChromoPainter/fineSTRUCTURE, in which each row and column represents one isolate and warmer colors indicate greater shared ancestry (higher co-ancestry) between strain pairs. Isolates are ordered according to the fineSTRUCTURE clustering tree shown above the matrix. The tree summarizes the hierarchical relationships among inferred clusters based on haplotype-sharing patterns. Colored annotation tracks indicate, from top to bottom, subtype assignment, geographic origin, and host ethnicity for each isolate

Analysis of the co-ancestry matrix indicated substantial heterogeneity within Chinese *H. pylori*. Six genetically distinct clusters were resolved, each showing a clear geographic pattern (Fig. 2). The largest group, designated SubtypeCentral (*n* = 461), represents the dominant lineage and is broadly distributed across central, eastern, and southwestern China. Isolates from this subtype were detected in Shanghai, Hunan, Chengdu, Shenzhen, Yunnan, Zhejiang, as well as in northern regions such as Heilongjiang and Beijing, suggesting that it captures a widespread mainland lineage.

Clear regional differentiation emerged outside this central group. In northern China, SubtypeInnerMongolia (*n* = 29) formed a distinct cluster that separated early from the continental core. These strains clustered phylogenetically closer to hpNorthAsia and hspSiberia reference lineages. Within this subgroup, 65% of isolates were obtained from hosts of Mongol ethnicity. In eastern China, SubtypeShandong (*n* = 215) appeared as a highly localized lineage. Nearly three-quarters of these isolates originated from Shandong Province, including strains from the Linqu area, a region with a high gastric cancer burden. This distribution suggests the presence of a geographically restricted lineage shaped by local evolutionary processes.

SubtypeSoutheast (*n* = 289) showed the strongest genetic affinity to the canonical hspEAsia population. Isolates in this group were mainly distributed along the southeastern coast, including Fujian, Hainan, and Shenzhen, and also included 48 strains originating from Taiwan. SubtypeYunnan comprised 136 isolates, of which 63 (46.3%) were sampled from Yunnan Province, while the remaining strains were distributed across multiple regions. This subtype included isolates from several ethnic minority populations, including Mosuo (*n* = 27), Pumi (*n* = 14), Naxi (*n* = 7), Bai (*n* = 3), and Tibetan (*n* = 1). In addition, seven hpSahul reference strains clustered within this group. SubtypeTaiwan (*n* = 45) formed a separate cluster that was clearly distinguishable from all other subtypes identified in this study. Additionally, 68 strains of Chinese origin were classified into non-HspEAsia reference strain lineages.

Phylogenetic reconstruction based on gene presence–absence matrix was largely concordant with the fineSTRUCTURE results (Figure S1). When rooted with global reference strains, Chinese isolates traced back to ancestral hpAfrica lineages, consistent with established models of *H. pylori* and human co-migration. Within this shared ancestry, pronounced diversification was observed across regions. Strains from Inner Mongolia and Yunnan showed evolutionary patterns distinct from those seen in most other provinces. In particular, Inner Mongolian isolates were closely related to hpSiberia and hpNorthAsia reference strains. This pattern indicates substantial genetic separation between northern, nomadic-associated populations and strains circulating in the Central Plains, consistent with long-term divergence or distinct ancestral contributions. Across 10 balanced down-sampled subsets, the six major fineSTRUCTURE-defined subpopulations remained consistently resolved, and PCA preserved the relative separation of the major subtype groups, supporting the robustness of the main clustering pattern. ADMIXTURE analysis provided convergent support for the six-group framework, with the lowest CV error (Figure S2). Under the GrafGen global classification scheme, most strains in the present cohort were assigned to hpgpAsia, indicating that the six Chinese subtypes identified here are best interpreted as finer-scale regional substructure within a broader Asian population background (Figure S3).

### Principal component analysis

**Fig. 3:**
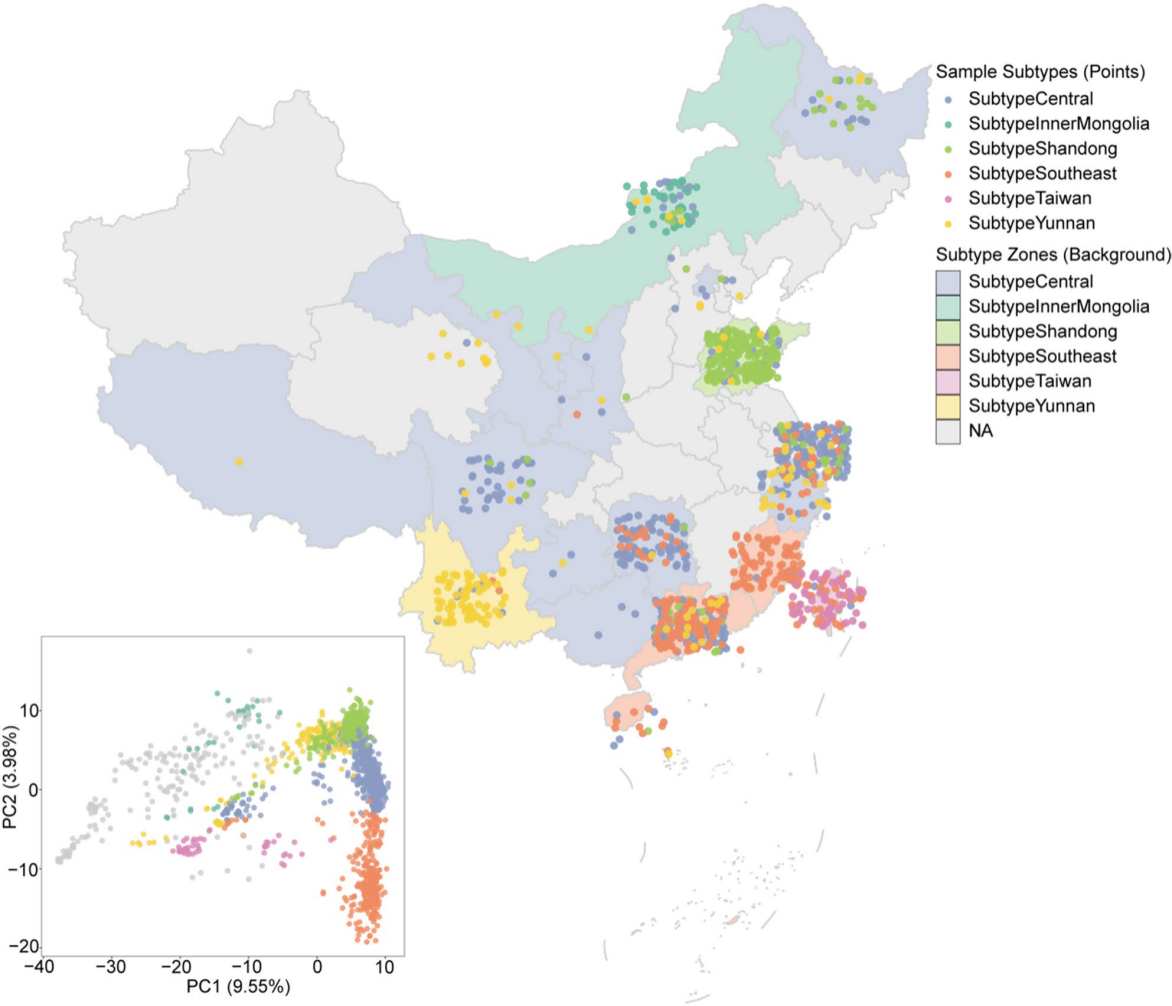
Principal component analysis of *H. pylori* strains included in this study. Each point represents one *H. pylori* isolate, colored according to the subtype assignment inferred by fineSTRUCTURE. Principal components 1 and 2 are shown on the x- and y-axes, respectively, and summarize the major axes of genomic variation in the Chinese dataset. The map displays the geographic origin of the isolates and the predominant subtype composition observed in different provinces or regions

To visualize the spatial distribution of genetic subpopulations, each strain was mapped according to its sampling location and colored by fineSTRUCTURE-defined subtype (Fig. 3). Overall, subtype assignments were broadly consistent with geographic origin. Regions characterized by high population mobility, including Shanghai, Zhejiang, Guangdong, and Hainan, showed clear patterns of subtype admixture. In Taiwan, SubtypeTaiwan and SubtypeSoutheast accounted for the majority of circulating strains, reflecting a distinct local population structure.

Principal Component Analysis further resolved genetic relationships among the identified subpopulations. The first two principal components captured major axes of variation that corresponded broadly to geographic longitude (PC1) and latitude (PC2). Most mainland subtypes, including SubtypeShandong, SubtypeCentral, and SubtypeYunnan, formed partially overlapping clusters, indicating shared genetic backgrounds. In contrast, SubtypeInnerMongolia and SubtypeTaiwan were positioned furthest from the main mainland cluster, consistent with their greater genetic differentiation. To formally evaluate the geographic interpretation of the PCA axes, we tested correlations between PCA and regional longitude and latitude. PC1 was primarily associated with longitude (R² = 0.436, *P* < 0.0001), whereas PC2 was most strongly associated with latitude (R² = 0.454, *P* < 0.0001), supporting an east–west pattern for PC1 and a north–south gradient for PC2 (Figure S4).

Comparison across regions showed that isolates from Yunnan and Inner Mongolia were more divergent from the central mainland population, whereas strains from central, eastern, and southern coastal regions exhibited tighter clustering and closer proximity to the mainland core. This pattern suggests higher genetic similarity among coastal and central populations, while peripheral regions retain more distinct genetic profiles.

### Ancestry analysis

**Fig. 4:**
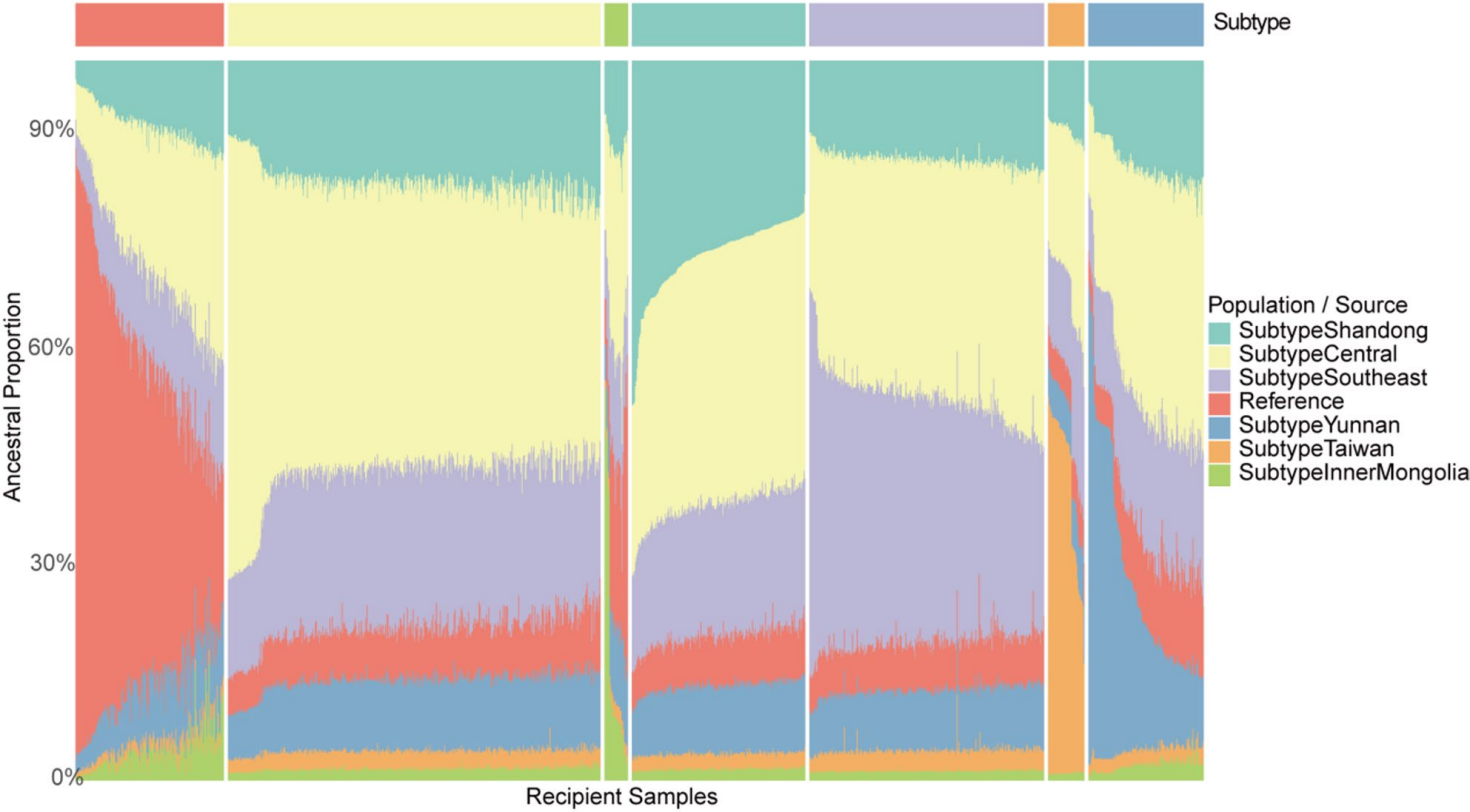
Fine-scale ancestry composition inferred by chromosome painting. Each stacked bar represents one recipient genome, grouped by subtype as indicated by the annotation bar above. Ancestry proportions were inferred by ChromoPainter using subtype-defined donor groups together with a global reference donor panel. Different colors within each bar denote the proportional contribution of the corresponding donor populations to the recipient genome, and the global “Reference” donor group is shown in salmon red. The y-axis indicates the fraction of painted ancestry assigned to each donor source (0–100%)

To characterize fine-scale ancestry patterns, chromosome painting was performed using members of each Chinese subtype together with a global “Reference” group as donors, and all remaining strains as recipients. This analysis revealed clear differences in ancestry composition among subpopulations (Fig. 4). SubtypeCentral, SubtypeSoutheast, and SubtypeShandong showed high levels of within-group ancestry, with most genomic segments assigned to donors from the same subtype. At the same time, these groups retained detectable contributions from other Chinese subtypes, including substantial SubtypeCentral ancestry in SubtypeShandong recipients.

In contrast, SubtypeTaiwan displayed a markedly distinct ancestry profile. Genomes from this subtype were almost entirely painted by SubtypeTaiwan donors, with minimal contributions from continental Chinese subtypes or the global reference group. This pattern remained consistent when alternative non–East Asian reference populations were used as donors and all other strains as recipients (Figure S5), indicating a stable and reproducible signal of genetic separation. SubtypeInnerMongolia and SubtypeYunnan exhibited more heterogeneous ancestry compositions. In SubtypeInnerMongolia, less than 30% of the genomic ancestry was derived from within the subtype itself, with substantial contributions from the global reference group as well as from SubtypeCentral and SubtypeSoutheast. SubtypeYunnan showed a similarly mixed profile, combining ancestry from its own subtype with notable input from SubtypeSoutheast and the reference population.

Taken together, these results show marked differences in ancestry structure across Chinese *H. pylori* subpopulations. Some groups, particularly SubtypeTaiwan, display strong within-lineage ancestry cohesion, whereas others, such as SubtypeInnerMongolia and SubtypeYunnan, reflect extensive admixture involving multiple ancestral sources.

### Pangenome characteristics and lineage-specific gene repertoires

**Fig. 5:**
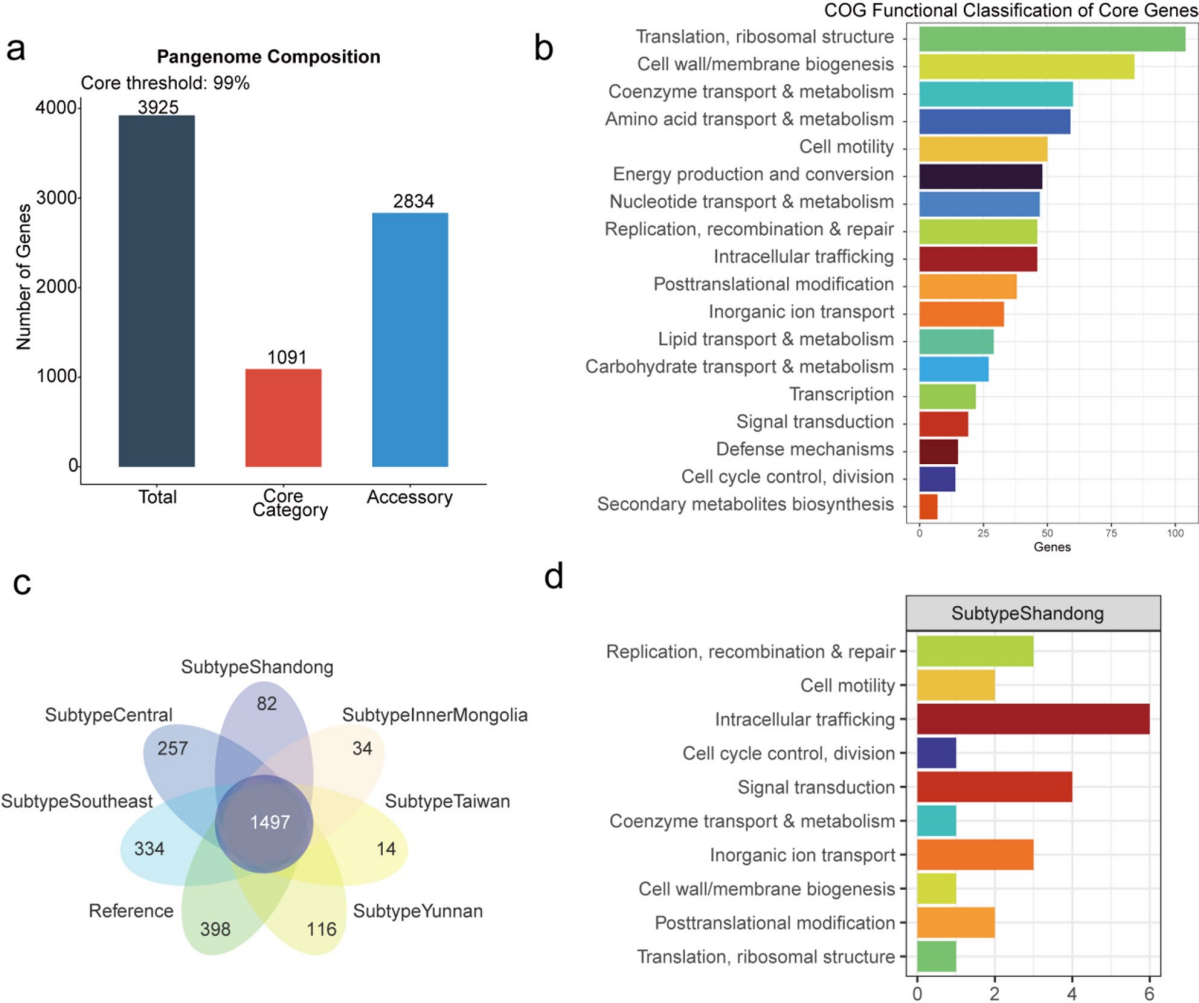
Pan-genome analysis of *H. pylori* strains (**a**) Composition of the pan-genome, showing the numbers of core and accessory gene clusters identified across all genomes. Core genes were defined as genes present in ≥ 99% of isolates. (**b**) Functional classification of the core genome based on COG categories after excluding genes without functional annotation. (**c**) Distribution of genes shared among the major subtype groups, illustrating the overlap between conserved and subtype-distributed gene repertoires. (**d**) Functional annotation of genes unique to SubtypeShandong, highlighting lineage-specific enrichment in selected biological processes

Pan-genome analysis identified a total of 3,925 non-redundant gene clusters across all *H. pylori* genomes. Among these, 1,091 genes were present in ≥ 99% isolates and were defined as the core genome, while the remaining 2,834 genes constituted the accessory genome (Fig. 5a). Functional annotation of the core genome, excluding genes with unknown function, indicated enrichment in essential cellular processes, including translation and ribosomal structure, cell wall/membrane biogenesis, and coenzyme transport & metabolism (Fig. 5b).

Comparison of gene repertoires across the identified subtypes showed that 1,497 genes were shared by all subpopulations and displayed functional profiles similar to those of the core genome (Fig. 5c). Subtype-specific gene sets were then examined to assess potential functional differentiation. In SubtypeShandong, a lineage prevalent in a region with high gastric cancer incidence, genes unique to this subtype were enriched in categories related to intracellular trafficking, signal transduction, and replication, recombination, and repair (Fig. 5d). In contrast, unique gene sets identified in the remaining subtypes showed broadly comparable functional distributions, without pronounced enrichment in specific categories (Figure S6a–d).

To further explore associations between *H. pylori* gene content and clinical presentation, genomes were grouped according to host disease status, including gastritis, gastric or duodenal ulcer, gastric cancer, and MALT lymphoma. Across these groups, 1,526 genes were shared (Figure S6e). After removal of genes with unassigned functions, the remaining genes were mainly involved in core biological processes such as translation, replication and repair, and cell wall or membrane biogenesis. In the gastric cancer group, 52 genes were identified as unique to this clinical category. These genes were primarily annotated to defense mechanisms and intracellular trafficking functions and showed functional profiles distinct from those observed in the other disease groups (Figure S6f).

### Divergent virulence profiles and local adaptation

**Fig. 6:**
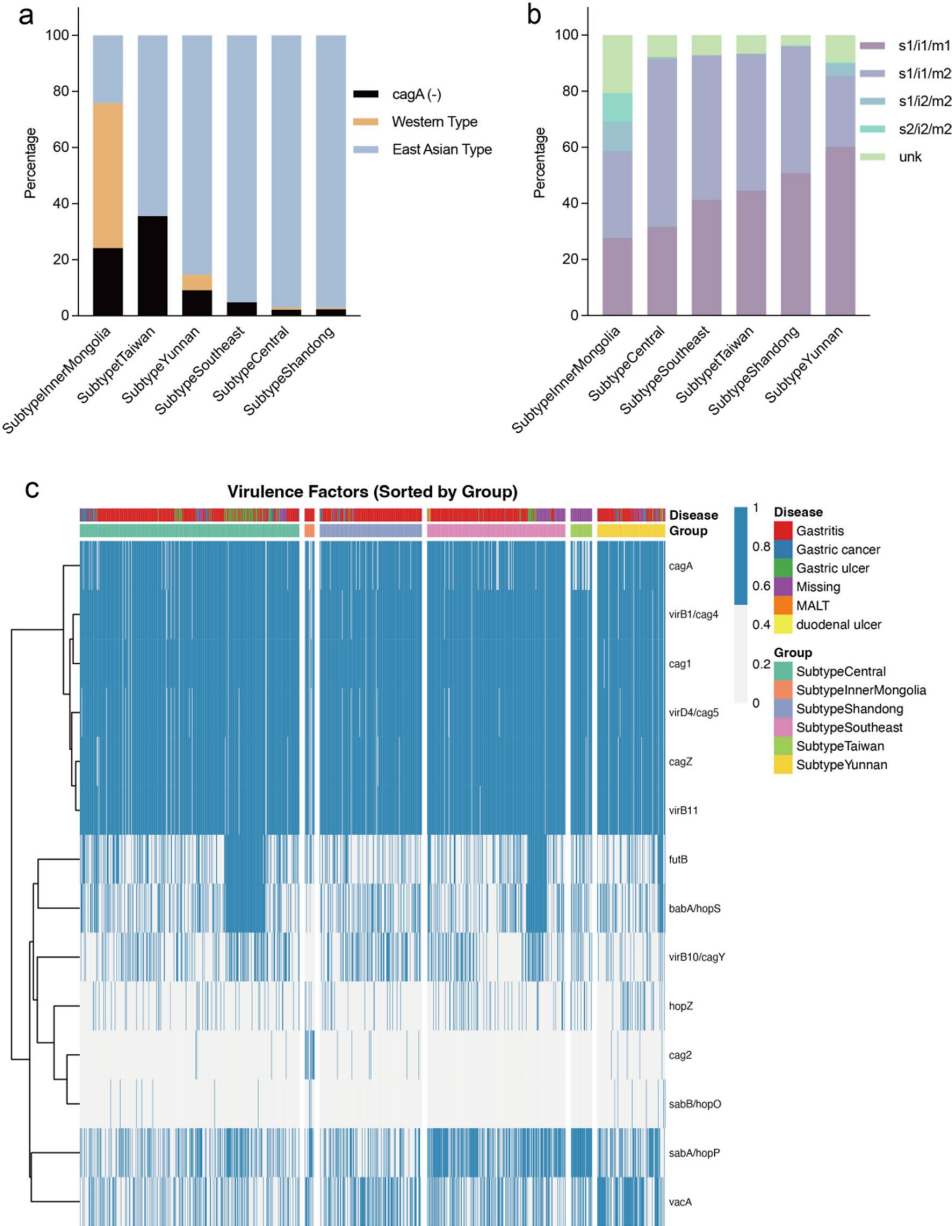
Distribution of virulence factors across *H. pylori* subtypes. (**a**) Distribution of cagA types across the six subtypes, classified according to EPIYA motif composition into East Asian-type, Western-type, or cagA-negative categories. (**b**) Distribution of vacA genotypes across subtypes, based on combinations of the signal (s), intermediate (i), and middle (m) regions. (**c**) Heatmap of virulence-associated genes showing subtype-level differences in prevalence. Only genes meeting the revised statistical criteria are displayed here (adjusted *P* < 0.05). Rows represent virulence-associated genes and columns represent individual isolates or grouped subtype summaries. Color intensity indicates gene presence/absence or relative prevalence

We examined the distribution of two major virulence determinants, *cagA* and *vacA*, across the defined subtypes. SubtypeInnerMongolia showed a higher proportion of *cagA*-negative strains and Western-type *cagA* variants than other subpopulations. A similar pattern, though less pronounced, was observed in SubtypeTaiwan. In contrast, SubtypeShandong, SubtypeSoutheast, and SubtypeCentral displayed largely uniform *cagA* profiles dominated by East Asian–type variants (Fig. 6a). Analysis of *vacA* genotypes revealed concordant patterns. SubtypeInnerMongolia harbored a heterogeneous combination of signal (s1/s2), intermediate (i1/i2), and middle (m1/m2) region alleles, whereas other Chinese subtypes showed more homogeneous *vacA* genotype distributions (Fig. 6b).

To assess broader differences in virulence gene content, all genomes were screened against the VFDB database. In total, 123 virulence-associated genes were detected, including genes located within the *cag* pathogenicity island, outer membrane protein genes (such as *hopS* and *hopT*), flagellar components (for example, *fliL* and *flgG*), urease-related genes (*ureA* and *ureB*), and enzymes involved in Lewis antigen synthesis (*futB* and *futC*). Among these, 11 virulence genes showed statistically significant differences in prevalence across the six subtypes (adjusted *P* < 0.05; Fig. 6c).

SubtypeInnerMongolia exhibited an overall lower prevalence of virulence genes, particularly those associated with the *cag* pathogenicity island. Given the high gastric cancer burden in Shandong Province, we further compared virulence gene frequencies between SubtypeShandong and the remaining subtypes (Figure S7). This analysis showed that *sabA/hopP* and *futB* were less commonly detected in this lineage after FDR correction.

### Genomic differentiation of *H. pylori* in China

**Fig. 7:**
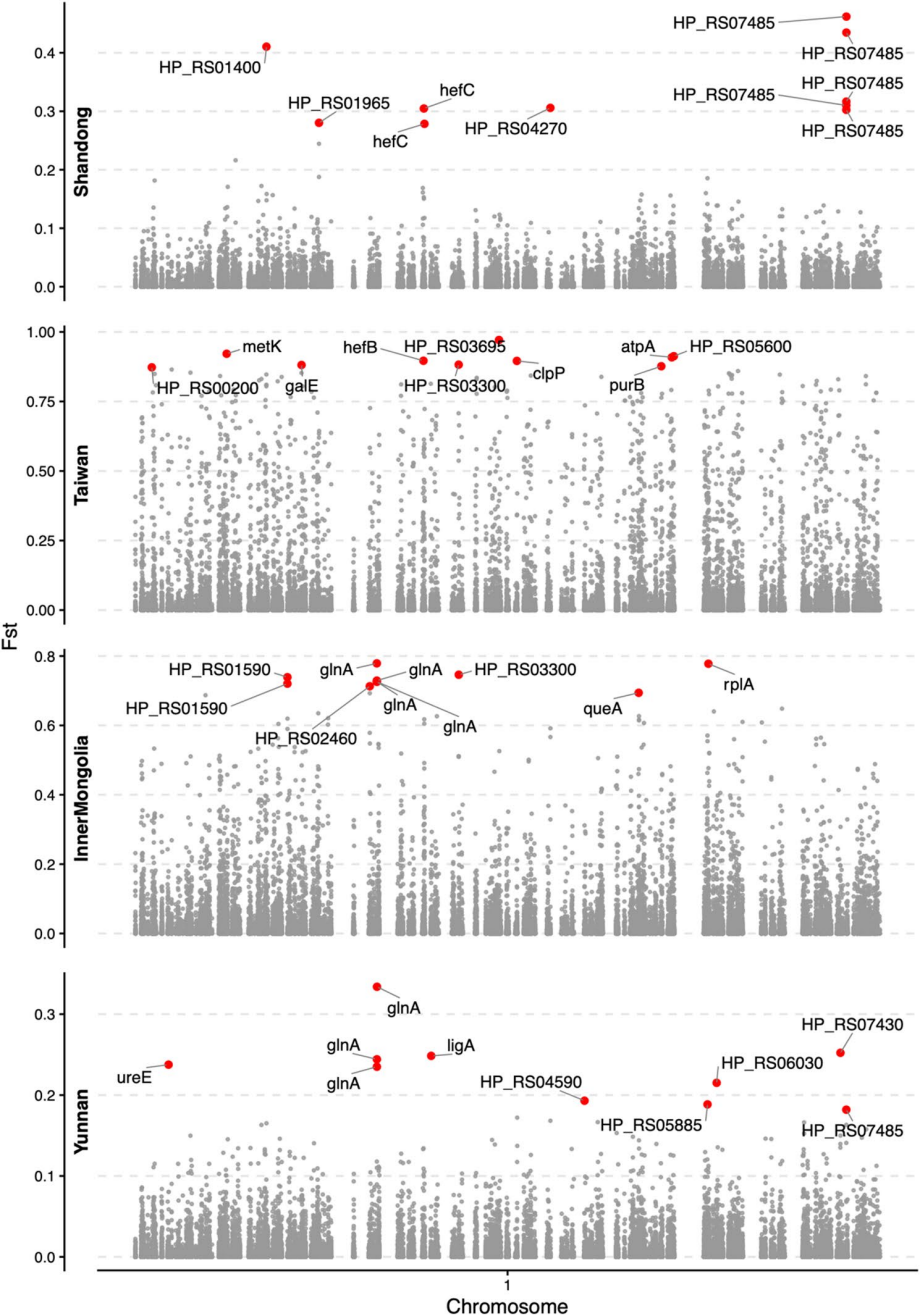
Fst analysis of genome-wide SNPs among *H. pylori* subpopulations in China. Each point represents one SNP, plotted according to its genomic position (x-axis) and Fst value (y-axis) in the comparison between a focal subtype and the remaining Chinese populations. For each subtype, the top-ranked SNPs with the highest Fst values are annotated with their corresponding gene names, including recurrent loci such as frpB4 (HP_RS07485), glnA, and HP1501 (HP_RS07430)

To assess genomic differentiation among *H. pylori* subpopulations in China, we performed Fst analysis based on genome-wide SNPs. For each subtype, the ten SNPs with the highest Fst values were examined in detail (Fig. 7). Overall, the distribution of Fst values differed markedly across subpopulations. SubtypeShandong and SubtypeYunnan showed relatively lower levels of differentiation from other Chinese populations, with most high-ranking SNPs exhibiting Fst values below 0.5. In SubtypeShandong, the top-ranking SNPs were mainly located in *HP_RS07485* (*frpB4*), *hefC*, and *HP_RS01400* (30 S ribosomal protein S1). In SubtypeYunnan, high-Fst SNPs were primarily mapped to *glnA*, *HP_RS07430* (*HP1501*), and *HP_RS07485* (*frpB4*).

In contrast, SubtypeInnerMongolia and SubtypeTaiwan displayed stronger genetic differentiation from other Chinese subpopulations. In the Taiwan subtype, SNPs with the highest Fst values were located in *HP_RS03695*, *metK*, *atpA*, and *hefB*. Elevated Fst values at *glnA* were also observed in SubtypeInnerMongolia. Across multiple subtypes, several loci repeatedly showed high Fst values, including *glnA*, *frpB4*, and *HP1501*. These loci therefore represent genomic regions that contribute disproportionately to population differentiation among Chinese *H. pylori* subpopulations.

### Genetic determinants of predicted antibiotic resistance

E-test MIC assays were performed for 50 newly collected clinical isolates. Metronidazole showed the highest resistance rate (96.0%, 48/50), followed by clarithromycin (44.0%, 22/50) and levofloxacin (36.0%, 18/50). Amoxicillin resistance was observed in 16.0% (8/50) of isolates, whereas resistance to rifampicin and tetracycline was rare (4.0% and 0%, respectively). Using phenotypic susceptibility as the reference, genotypic predictions were most accurate for metronidazole (98%) and levofloxacin (91.7%), with substantial (κ = 0.785) and almost perfect (κ = 0.822) agreement. Using phenotypic susceptibility as the reference, genotype-based prediction showed variable performance across antibiotics. Agreement was strongest for metronidazole and levofloxacin, was moderate for clarithromycin and amoxicillin, and was limited for rifampicin because only two phenotypically resistant isolates were observed. Tetracycline could not be meaningfully evaluated because no resistant isolate was detected in this validation subset (Table 1). These results support the utility of mutation-based prediction as a population-level surveillance tool, while also highlighting its antibiotic-specific limitations.

Genomic analysis revealed marked geographic heterogeneity in predicted antibiotic resistance profiles across *H. pylori* subtypes in China (Table 2). Elevated resistance to levofloxacin and metronidazole was observed in northern subpopulations, particularly within the Shandong and Inner Mongolia subtypes. Among all groups, SubtypeInnerMongolia showed the highest prevalence of resistance to amoxicillin (20.69%) and tetracycline (6.90%), as well as the highest proportion of multidrug-resistant (MDR) strains (34.48%). In contrast, rifampicin resistance displayed a distinct regional pattern. The SubtypeTaiwan lineage (*n* = 45) exhibited a markedly higher predicted rifampicin resistance rate (60.00%) than all other subpopulations (*P* < 0.001), representing the most pronounced resistance disparity observed in this dataset.

At the molecular level, resistance-associated hotspot mutations showed strong concordance with predicted resistance phenotypes (Table S4 and S5). Levofloxacin resistance was mainly associated with nonsynonymous substitutions at positions N87 and D91 in *gyrA*, as well as the D481E mutation in *gyrB*. The high level of rifampicin resistance observed in the Taiwan subtype was almost exclusively attributable to the *rpoB* A2414V mutation (*HP_RS05885*), which was detected in 60.00% of isolates from this group compared to other lineages (< 1%) (*P* < 0.001). Metronidazole resistance was primarily linked to the *rdxA* S108A mutation, which occurred at high frequency in subpopulations with elevated resistance rates. Together, these findings demonstrate that predicted resistance profiles across Chinese *H. pylori* subtypes are underpinned by subtype-specific genetic variants.

**Table 1:** Comparison of phenotypic resistance detected by E-test with predicted genotypic resistance in 50 clinical strains

Antibiotic	Phenotypic *R*, *n* (%)	Genotypic S, *n*	Genotypic *R*, *n*	Accuracy (%)	Sensitivity (%)	Specificity (%)	PPV (%)	NPV (%)	Kappa
AML	8 (16.0)	42	8	88.0	62.5	92.9	62.5	92.9	0.554
CLR	22 (44.0)	33	17	86.0	72.7	96.4	94.1	81.8	0.709
LEV	18 (36.0)	34	16	92.0	83.3	96.9	93.8	91.2	0.822
MTZ	48 (96.0)	3	47	98.0	97.9	100.0	100.0	66.7	0.790
RFP	2 (4.0)	46	4	88.0	0.0	91.7	0.0	95.7	-0.056
TET	0 (0.0)	50	0	100.0	—	100.0	—	100.0	0

AML amoxicillin; CLR clarithromycin; LEV levofloxacin; MTZ metronidazole; RFP rifampicin; TET tetracycline; PPV, positive predictive value; NPV, negative predictive value

**Table 2:** Distribution of predicted antibiotic resistance among *H. pylori* strains across subtypes

Antibiotic	SubtypeCentral	SubtypeInnerMongolia	SubtypeShandong	SubtypeSoutheast	SubtypeTaiwan	SubtypeYunnan	*P*	Adjusted *P*
	(*N* = 461)	(*N* = 29)	(*N* = 215)	(*N* = 289)	(*N* = 45)	(*N* = 136)		
AML	53 (11.50%)	6 (20.69%)	23 (10.70%)	35 (12.11%)	4 (8.89%)	18 (13.24%)	0.68	0.79
CLR	166 (36.01%)	14 (48.28%)	92 (42.79%)	81 (28.03%)	5 (11.11%)	41 (29.93%)	0.0001	0.0002
LEV	155 (33.62%)	12 (41.38%)	96 (44.65%)	67 (23.18%)	4 (8.89%)	43 (30.15%)	0.00005	0.0001
MTZ	427 (92.62%)	28 (96.55%)	202 (93.95%)	271 (93.77%)	43 (95.56%)	125 (91.91%)	0.931	0.931
RFP	37 (8.03%)	5 (17.24%)	12 (5.58%)	20 (6.92%)	27 (60%)	22 (16.18%)	0.00005	0.0001
TET	5 (1.08%)	2 (6.90%)	9 (4.19%)	1 (0.35%)	0 (0.00%)	4 (2.94%)	0.00337	0.0049
MDR	108 (23.43%)	10 (34.48%)	64 (29.77%)	44 (15.22%)	7 (15.56%)	37 (27.20%)	0.00078	0.0018

AML amoxicillin; CLR clarithromycin; LEV levofloxacin; MTZ metronidazole; RFP rifampicin; TET tetracycline

## Discussion

In East Asia, *H. pylori* populations are often grouped under the single label of hspEAsia [33], a framework that emphasizes shared ancestry and generally elevated virulence potential. However, within China, gastric cancer incidence varies strikingly across regions [34], likely reflecting the combined effects of host, environmental, dietary, healthcare, and microbial factors. By analyzing more than 1,200 *H. pylori* genomes collected from 20 provinces and regions, including newly sequenced isolates from Shanghai, we show that Chinese hspEAsia strains contain substantial internal structure rather than forming a single homogeneous population. Instead, they form six geographically structured subpopulations with distinct evolutionary histories, gene repertoires, virulence profiles, and antibiotic resistance patterns. This refined population framework provides a basis for investigating whether bacterial population structure contributes to regional heterogeneity in disease burden within China. Although sampling density was uneven across provinces, this study covers 20 provinces and regions across China and, importantly, fills genomic data gaps in previously underrepresented frontier and high-latitude areas. Inclusion of these regions is critical for resolving the structure of peripheral *H. pylori* populations within China.

Large-scale analyses focused specifically on *H. pylori* population structure within China have been limited. Earlier studies either concentrated on restricted geographic areas or placed Chinese strains within broader pan–East Asian frameworks without resolving internal structure [12, 35]. For example, global analyses [9] identified multiple hspEAsia branches originating from China but did not define lineage boundaries within the country. Regional surveys [8, 36] suggested genetic clustering in provinces such as Fujian, northeastern China, or Yunnan, yet limited sample sizes precluded comprehensive resolution. Our findings are broadly consistent with these earlier observations but extend them by resolving additional, previously underdefined lineages, including those predominant in Shandong and Taiwan. By integrating fine-scale ancestry inference with pangenome composition, virulence determinants, and resistance-associated mutations, our study reveals a level of within-China diversification that was not apparent in earlier work.

The geographic structure of Chinese *H. pylori* subpopulations mirrors major patterns of human demographic history in East Asia. The genetic affinity of strains from Inner Mongolia to hpNorthAsia and Siberian lineages is consistent with long-standing human interactions across the Eurasian steppe, a region known to have facilitated repeated waves of migration and admixture [37]. Similarly, the distinct composition of the Yunnan subtype aligns with evidence from ancient and modern human genomics showing deeply differentiated ancestry in this region, shaped by interactions among Tibetan Plateau populations, southern East Asians, and Southeast Asian groups [38]. In contrast, the widespread distribution of SubtypeCentral, SubtypeShandong, and SubtypeSoutheast parallels the historical expansion of Han Chinese populations across central, eastern, and coastal China [39]. The Taiwan subtype reflects a more complex history, combining contributions from mainland-derived lineages linked to southeastern China with signals consistent with longer-term island-specific evolution [40]. Together, these patterns support the view that *H. pylori* diversity in China has been shaped by both human migration and long-term regional separation.

Signals of genomic differentiation further suggest that demographic history alone does not fully explain the observed population structure. Several loci repeatedly exhibited elevated Fst values across multiple subpopulations, including *glnA*, *frpB4*, and *HP1501*. These genes are functionally linked to processes central to *H. pylori* persistence, such as nitrogen metabolism, iron acquisition, and outer membrane function. Variation in *glnA* may influence bacterial fitness under fluctuating nutrient and immune conditions in the gastric niche [41], while divergence in *frpB4*, a component of TonB-dependent transport, is consistent with adaptation to region-specific constraints on iron availability [42, 43]. Similarly, outer membrane proteins such as HP1501 may modulate interactions with the host environment, including immune recognition or antibiotic permeability [44]. The repeated involvement of these loci across geographically distinct subtypes suggests that local selective pressures acting on metabolic capacity and membrane-associated functions contribute to ongoing diversification within Chinese *H. pylori* populations. Although small subpopulation sizes may increase the influence of genetic drift, the highly differentiated sites identified in this study were primarily concentrated in genes with well-defined functions, such as the iron transport gene frpB4 and the nitrogen metabolism gene glnA, rather than being randomly distributed across the genome.

Differences in virulence gene composition across subtypes may be relevant to regional heterogeneity in gastric cancer burden, but the current ecological and cross-sectional analyses do not establish subtype-specific cancer risk. Although hspEAsia strains are often regarded as uniformly high-risk, our data show substantial heterogeneity in the distribution of key virulence determinants. Peripheral subtypes, particularly those from Inner Mongolia, exhibited a higher prevalence of *cagA*-negative strains or Western-type *cagA* variants, together with more diverse *vacA* genotypes and a reduced overall prevalence of genes within the cag pathogenicity island. These features are generally associated with attenuated inflammatory responses and lower oncogenic potential [45–47]. In contrast, the Shandong subtype, which predominates in a well-recognized high-incidence region, was enriched for *sabB*, *hopH* (OipA), and *cagY*, while showing reduced prevalence of *sabA*. These genes represent biologically plausible candidate factors linked to epithelial interaction and inflammatory signaling [48–51]. Importantly, these observations do not imply direct causality but indicate that regional *H. pylori* populations differ systematically in virulence-related gene content.

Predicted antibiotic resistance profiles also differed markedly among subtypes, reinforcing the view that contemporary selective pressures interact with lineage background [52, 53]. Previous large-scale studies have demonstrated that whole-genome sequencing–based prediction of clarithromycin and levofloxacin resistance using mutations in 23S rRNA and gyrA achieves both sensitivity and specificity exceeding 95%. Accordingly, genotype-based prediction provides a reliable surrogate for assessing resistance potential in large *H. pylori* populations [54, 55]. Resistance to levofloxacin and metronidazole was concentrated in northern subpopulations, whereas the Taiwan subtype displayed an exceptionally high prevalence of predicted rifampicin resistance driven almost entirely by a single *rpoB* mutation. This lineage-enriched pattern may reflect local selective pressures, founder effects, and/or expansion of strains carrying this mutation [56]. An additional pattern of interest was observed in the Inner Mongolia subtype, which showed a comparatively lower prevalence of several virulence-associated markers but a higher frequency of predicted multidrug resistance. Multiple non-mutually exclusive explanations may contribute to this pattern, including small subgroup size, regional sampling structure, host demographic background, lineage history, and local antimicrobial selection pressures. Beyond the canonical target-gene model, emerging work suggests that MDR in *H. pylori* can also involve broader physiological routes, including mutations in lipid metabolism such as fabH (e.g., A149G), which have been proposed to contribute to multi-drug phenotypes [57]. More broadly, the resistance-associated mutations identified in this study align well with established molecular mechanisms, supporting the reliability of genome-based resistance inference while highlighting the uneven geographic distribution of resistance determinants within China.

We demonstrate that Chinese *H. pylori* comprises regionally structured subpopulations with subtype-dependent resistance determinants and distinct virulence genes. These findings may have potential translational relevance for future surveillance and molecular epidemiology studies. Current *H. pylori* clinical management still relies largely on empirical treatment. The 2022 Chinese national clinical practice guideline emphasizes tailoring regimens according to prior antibiotic exposure and does not recommend routine susceptibility testing for initial therapy [58]. Consequently, most patients begin treatment without strain-level information, and repeated treatment attempts may accumulate following eradication failure [59]. Furthermore, indications for *H. pylori* treatment are still being debated [60], and risk-stratified or individualized treatment strategies are not routinely implemented in clinical practice [61]. Incorporating subtype-based stratification into diagnostic workflows could strengthen surveillance and follow-up of *H. pylori* infection and provide a genomic basis for more targeted and personalized treatment selection.

Several limitations warrant consideration. Sampling density varied across regions, and some provinces were represented by relatively few isolates, which may limit the detection of rare local lineages. To assess the robustness of the major clustering framework, we performed balanced down-sampling sensitivity analyses across 10 independent random subsets, which supported the stability of the six-subpopulation structure. In addition, antibiotic resistance was inferred from genomic data rather than measured phenotypically. To provide supportive phenotypic context, we performed E-test MIC assays in the 50 newly collected clinical isolates and observed overall concordance with mutation-based predictions. Future work integrating subtype assignment with patient-level outcomes, antimicrobial susceptibility testing, and prospective validation will be required before subtype-aware risk stratification or treatment guidance can be considered for clinical use.

In conclusion, by resolving Chinese hspEAsia *H. pylori* into six geographically structured subpopulations, this study demonstrates substantial genetic, functional, virulence-related, and predicted resistance heterogeneity within what is often treated as a single lineage. These results emphasize the importance of considering population substructure when interpreting regional heterogeneity in gastric cancer burden and predicted antibiotic resistance patterns, and they provide a genomic framework for future evaluation of region-aware surveillance strategies.

## Electronic Supplementary Material

Below is the link to the electronic supplementary material.

Supplementary Material 1

Supplementary Material 2

## Data Availability

The sequencing data from this study are publicly available at NCBI at https://www.ncbi.nlm.nih.gov/bioproject/PRJNA1217501. (BioProject ID PRJNA1217501).

## Abbreviations

AML: Amoxicillin
ANI: Average Nucleotide Identity
ASR: Age-standardized rate
cagA: Cytotoxin-associated gene A
CLR: Clarithromycin
COG: Clusters of Orthologous Groups
DNA: Deoxyribonucleic acid
EPIYA: Glu–Pro–Ile–Tyr–Ala motif
EPIYA-ABC: Western-type CagA EPIYA pattern
EPIYA-ABD: East Asian-type CagA EPIYA pattern
FASTQ: Sequencing read format (FASTQ file)
Fst: Fixation index
FUSCC: Fudan University Shanghai Cancer Center
GC: Gastric cancer
H. pylori: Helicobacter pylori
iTOL: Interactive Tree Of Life
LEV: Levofloxacin
MCMC: Markov chain Monte Carlo
MDR: Multidrug-resistant
MTZ: Metronidazole
NCBI: National Center for Biotechnology Information
QUAST: Quality Assessment Tool for Genome Assemblies
RFP: Rifampicin
SNP: Single-nucleotide polymorphism
SPAdes: St. Petersburg genome assembler
TET: Tetracycline
vacA: Vacuolating cytotoxin A
VFDB: Virulence Factor Database
