# Supplementary material for: Genomic landscape and fine-scale population structure of Helicobacter pylori across China
Source: Biomark Res. 2026 May 16;14:66. doi: 10.1186/s40364-026-00932-0 (PMC13330326; doi:10.1186/s40364-026-00932-0)
Supplement: Supplementary file 1 — Supplementary Material 1 [file 40364_2026_932_MOESM1_ESM.docx]

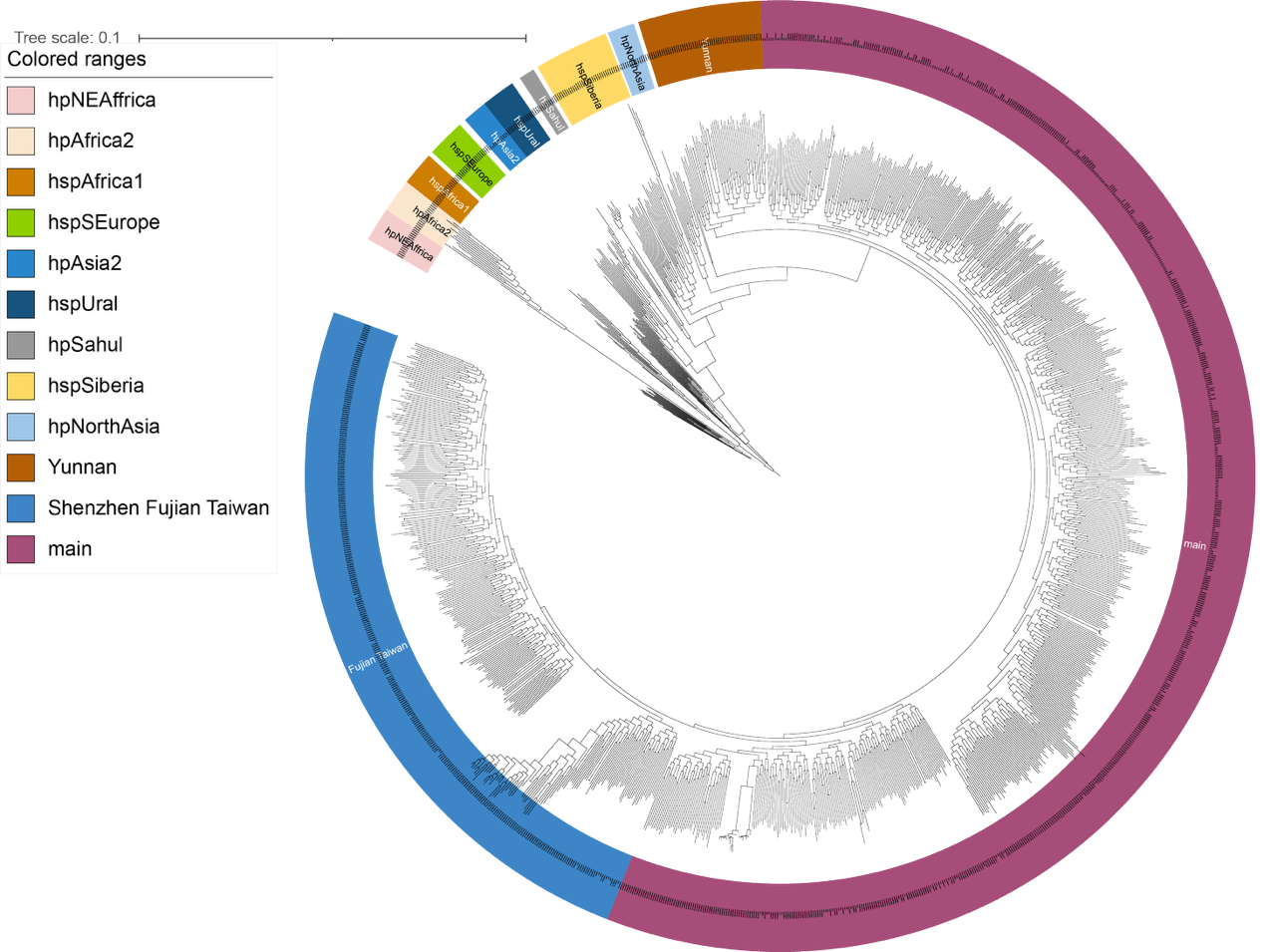


**Figure S1. Phylogenetic tree was constructed from the gene presence–absence matrix.** The tree was constructed from the gene presence–absence matrix generated by Panaroo and visualized to compare the accessory-genome–based relationships with the fineSTRUCTURE-defined subtype framework. Major clades corresponding to central/western mainland China and peripheral regional groups are indicated.

**Figure S2. CV error plot from the cross-validation procedure in ADMIXTURE to infer the best number of clusters.**

**Figure S3. Genetic distance by GrafGen analysis.** Most strains in the present cohort were assigned to hpgpAsia based on classification of 1,011 HpGP sequences from 50 countries, indicating that the six Chinese subtypes identified here are best interpreted as finer-scale regional substructure within a broader Asian population background.


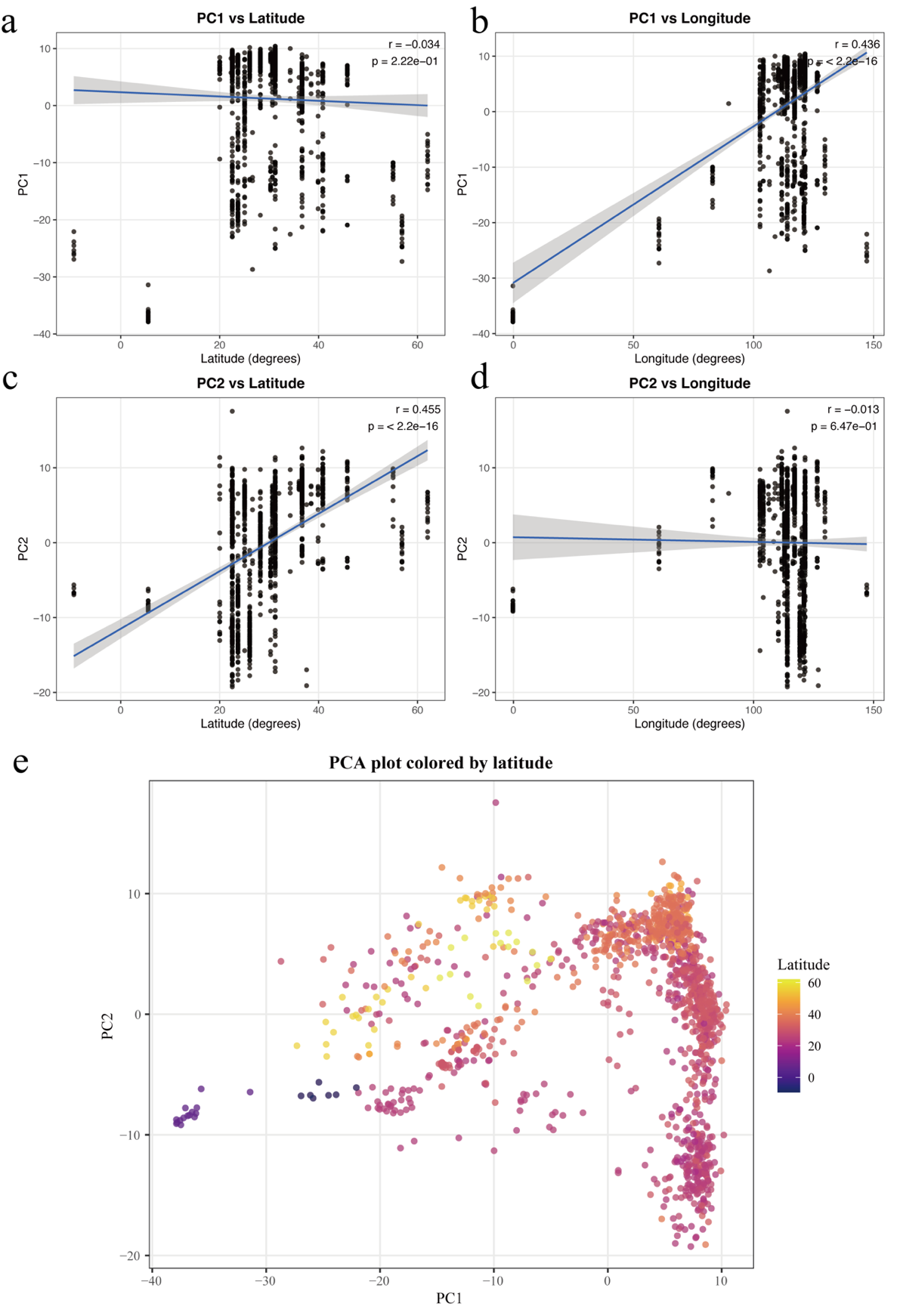


**Figure S4. Association between principal components and geographic variables.** (a–d) Scatter plots showing the relationships of PC1 and PC2 with longitude and latitude. Each point represents one *H. pylori* isolate. Solid lines indicate linear regression fits with 95% confidence intervals. Pearson’s correlation coefficients (r) and two-sided p values are shown in each panel. (e) PCA plot (PC1 vs PC2) colored by sample latitude, illustrating the geographic gradient across isolates.


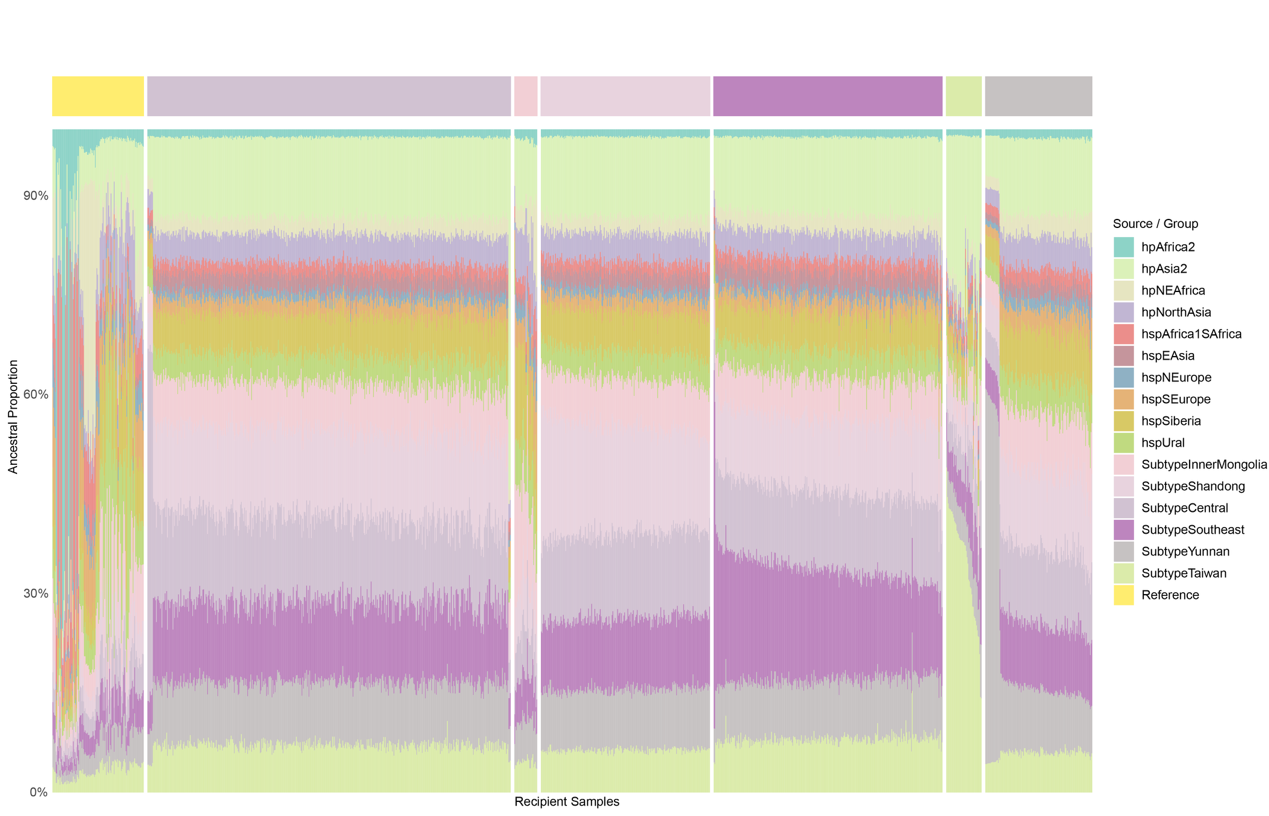


**Figure S5. Ancestral chromosome mapping analysis.** Reference genomes from different global populations, including hpAfrica2, hpAfrica1, hpNEAfrica, hpAsia2, hpNorthAsia, hspUral, and hspEAsia, were used as donors to test the stability of ancestry inference. The resulting ancestry profiles support the distinct ancestry composition of the major Chinese subtype groups, particularly the relative separation of SubtypeTaiwan.


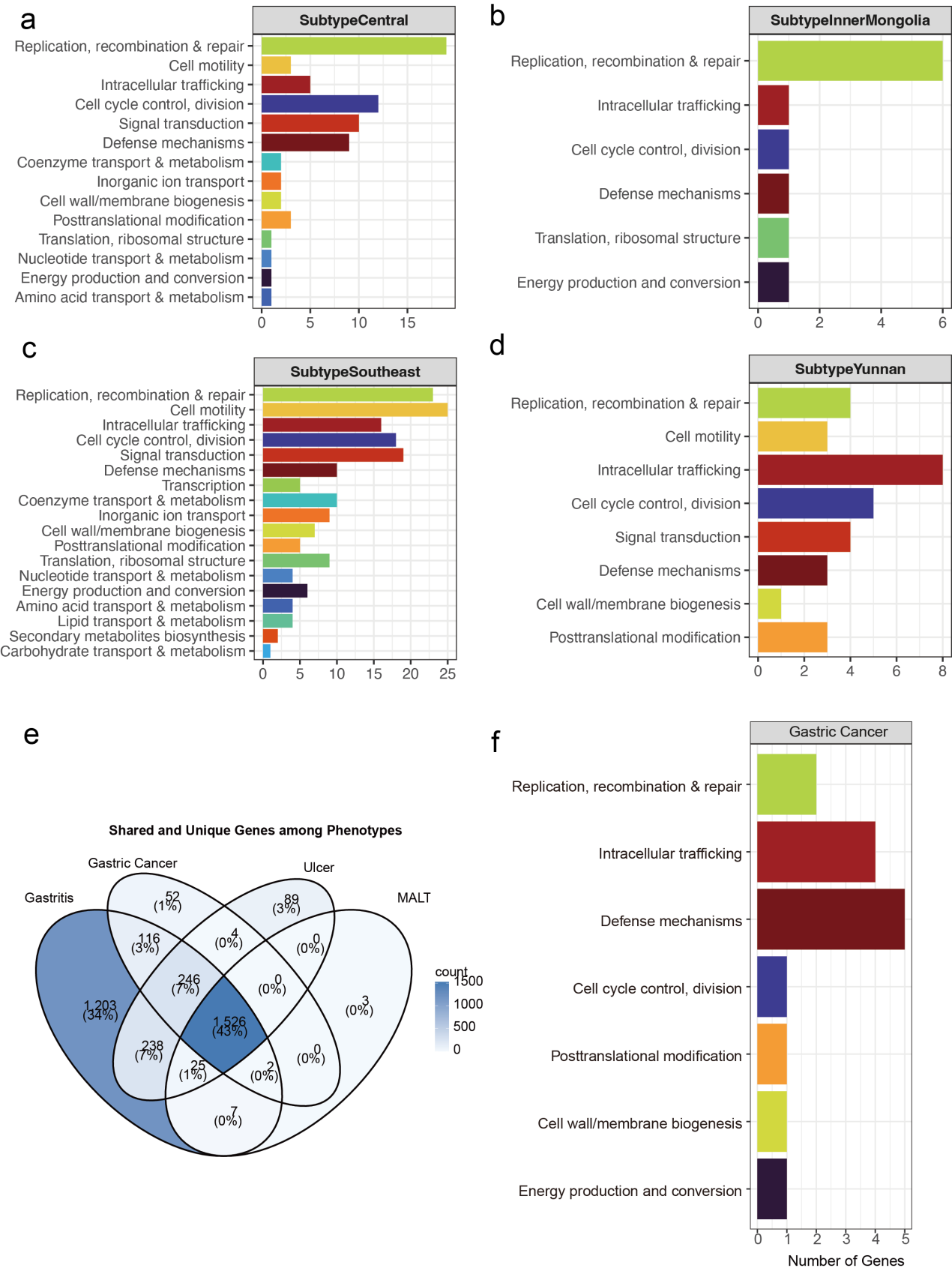


**Figure S6. Subtype-specific and disease-specific genes.** (a–d) Functional annotation of subtype-specific genes for selected subtype groups. (e) Distribution of shared and phenotype-specific genes across clinical disease categories. (f) Functional annotation of genes unique to the gastric cancer group. SubtypeTaiwan was not annotated in panels (a–d) because the number of subtype-specific genes was too small for stable functional summarization (n = 14).

**Figure S7. Heatmap of differential virulence factors.** The heatmap compares the prevalence of virulence-associated genes between SubtypeShandong and all other Chinese subtype groups combined. Annotation bars indicate disease phenotype and group labels (adjusted P < 0.05).
